# Routine screening of emergency admissions at risk of chronic hepatitis (SEARCH) identifies and links hepatitis B cases to care

**DOI:** 10.1111/liv.15414

**Published:** 2022-09-14

**Authors:** Rachael Jacob, David S. Prince, Joseph L. Pipicella, Angela Nguyen, Melissa Bagatella, Frank Alvaro, Michael Maley, Hong Foo, Paul Middleton, Tahrima Kayes, Julia DiGirolamo, Scott A. Davison, Miriam T. Levy

**Affiliations:** ^1^ Department of Gastroenterology and Liver Liverpool Hospital Sydney New South Wales Australia; ^2^ The University of New South Wales Sydney New South Wales Australia; ^3^ The Ingham Institute for Applied Medical Research Liverpool New South Wales Australia; ^4^ School of Medicine Western Sydney University Penrith New South Wales Australia; ^5^ New South Wales Health Pathology Sydney New South Wales Australia; ^6^ South Western Emergency Research Institute, UNSW Sydney New South Wales Australia

**Keywords:** hepatitis B, linkage to care, universal screening

## Abstract

**Background and Aims:**

Significant barriers exist with hepatitis B (HBV) case detection and effective linkage to care (LTC). The emergency department (ED) is a unique healthcare interaction where hepatitis screening and LTC could be achieved. We examined the efficacy and utility of automated ED HBV screening for Overseas Born (OB) patients.

**Methods:**

A novel‐automated hepatitis screening service “SEARCH” (Screening Emergency Admissions at Risk of Chronic Hepatitis) was piloted at a metropolitan hospital. A retrospective and comparative analysis of hepatitis testing during the SEARCH pilot compared to a period of routine testing was conducted.

**Results:**

During the SEARCH pilot, 4778 OB patients were tested for HBV (86% of eligible patient presentations), compared with 1.9% of eligible patients during a control period of clinician‐initiated testing. SEARCH detected 108 (2.3%) hepatitis B surface antigen positive patients including 20 (19%) in whom the diagnosis was new. Among 88 patients with known HBV, 57% were receiving medical care, 33% had become lost to follow‐up and 10% had never received HBV care. Overall, 30/88 (34%) patients with known HBV were receiving complete guideline‐based care prior to re‐engagement via SEARCH. Following SEARCH, LTC was successful achieved in 48/58 (83%) unlinked patients and 19 patients were commenced on anti‐viral therapy. New diagnoses of cirrhosis and hepatocellular carcinoma were made in five and one patient(s) respectively.

**Conclusions:**

Automated ED screening of OB patients is effective in HBV diagnosis, re‐diagnosis and LTC. Prior to SEARCH, the majority of patients were not receiving guideline‐based care.

**Abbreviations**
HBV: hepatitis B
LTC: linkage to care
ED: emergency department
OB: overseas born
SEARCH: Screening of Emergency Admissions at Risk of Chronic Hepatitis
HBsAg: hepatitis B surface antigen
LTFU: lost to follow‐up
AVT: antiviral therapy
HCC: hepatocellular carcinoma
WHO: World Health Organisation
HCV: hepatitis C
PWID: persons who inject drugs
ATSI: Aboriginal or Torres Strait Islander peoples
CALD: culturally and linguistically diverse
PCP: primary care physician
HBeAg: hepatitis B e antigen
HDV: hepatitis D
APRI: aminotransferase to platelet ratio index
($AUD): Australian dollars
CP: Child Pugh
BBV: blood‐borne viruses

**Lay Summary**

- Australians living with chronic hepatitis B (HBV) are frequently undiagnosed or not receiving appropriate care. Clinician initiated uptake of risk‐factor‐based screening in the emergency department remains low.
- A pilot service (SEARCH) of automated and routine hepatitis screening of high‐risk groups in the emergency department (ED) was successful in HBV diagnoses, re‐diagnoses and linkage to care.
- Routine, automated and targeted HBV ED screening is effective and could address gaps in HBV care.

## 1 INTRODUCTION

Hepatitis B (HBV) is a global public health challenge with chronic infection leading to cirrhosis and hepatocellular carcinoma (HCC). Early diagnosis and antiviral therapy (AVT) are fundamental to reducing liver‐related morbidity and mortality.^1^ The World Health Organisation (WHO) prioritises HBV, aiming to reduce mortality by 65% by 2030.^1^ Such an ambitious goal will require significant public health innovation.^2^ Hepatitis C (HCV) micro‐elimination strategies in prison and in persons who inject drugs (PWID) have been efficacious.^3^, ^4^ Micro‐elimination strategies for HBV will differ and require consideration of where these patients are most likely to be found epidemiologically.

The Australian healthcare system delivers high rates of HBV vaccination and excellent maternal and perinatal care; however, gaps exist in diagnosis and linkage to care (LTC) of people with existing chronic infection.^5^ Whilst the Australian National Testing Policy endorses testing priority populations, including those born in high prevalence countries and Aboriginal or Torres Strait Islander (ATSI) peoples,^6^ the uptake remains poor. It is estimated that up to 30% of HBV‐infected individuals remain undiagnosed in Australia.^6^

The majority of people living with chronic HBV in Australia are overseas born (OB).^5^ Many acquired the infection in childhood and may be unaware of their status. Those from culturally and linguistically diverse backgrounds (CALD) face barriers engaging with health promotion material but do attend the emergency department (ED) as necessary.^7^ Hence, the ED represents a potential opportunity to engage.

It has been shown that screening for chronic HBV is cost‐effective, particularly in high‐risk populations.^8^, ^9^, ^10^ Universal screening is accepted in obstetric populations and could be implemented in other populations when aligned with the National Testing Policy. A call for universal HBV testing in Australia has been made to reduce complexity in decision making,^11^ however, barriers to implementation will remain. Despite policy, healthcare workers may fail to implement screening, as the patient's presenting healthcare concern often takes priority. We considered the ED to be a place where an automated and universal approach could be implemented for testing the target population.

This study aims to assess the Screening of Emergency Admissions at Risk of Chronic Hepatitis (SEARCH) pilot—efficacy (testing rates) and utility (infection rates and LTC) of automated ED screening in OB patients and compare this to a period of routine clinician‐initiated testing. During the pilot service, patient selection by their demographics triggered hepatitis testing utilising serum samples already collected as part of their health care assessment in the ED. A cost analysis was also performed to describe the total actual costs of this pilot service. It also aimed to report the cost per patient tested and per hepatitis B surface antigen (HBsAg) positive patient identified.

## 2 METHODS

### 2.1 Description of SEARCH pilot

An automated screening service entitled SEARCH was a single centre pilot conducted in an Australian metropolitan hospital ED. SEARCH tested 4778 OB patients for HBsAg. Patients presenting to ED routinely report demographic information including country of birth. Using a computer algorithm, OB patients aged 18–80 years were identified and tested. Patients were not tested if they (i) withdrew consent for hepatitis testing (ii) had insufficient serum samples to allow HBsAg testing or (iii) died during the ED presentation. Robotically retrieved biochemistry serum samples were tested for HBsAg using electrochemiluminescent microparticle immunoassay (Elecsys HBsAg II, Roche Diagnostics cobas®). ATSI patients were also tested for the pilot service in accordance with the National Testing Policy, however, their data was not examined in this retrospective analysis.

Consent for the hepatitis testing during SEARCH utilised information provided on multilingual educational posters and brochures, displayed in prominent areas of the ED. Emergency medical staff advised patients of hepatitis testing at the time of provision of information about all blood tests being performed. Patients could withdraw consent for hepatitis testing by informing emergency staff or via a 24‐hour mobile number.

HBV diagnoses were considered *new* if (i) there was no prior record of a positive HBsAg within networked hospital laboratory or external pathology records and (ii) both patient and nominated primary care physician (PCP) were unaware. Patients were considered *lost to follow‐up* (LTFU) if they had previously seen a PCP or specialist for HBV but were not currently receiving HBV care.

HBV care milestones prior to LTC through SEARCH were assessed as being *complete* or *incomplete*, based on international guidelines.^12^, ^13^ Complete HBV care was defined as (i) HBV DNA quantification within 12 months (ii) on AVT if treatment was indicated and (iii) participation in HCC screening if indicated within prior 8 months.

LTC for HBsAg positive patients identified through SEARCH was provided by direct meeting by liver clinic staff with those who were still inpatients. If already discharged, the patient's PCP was contacted and if unavailable, the patient was contacted directly. Active re‐engagement during subsequent hospital presentations was attempted if patients were unable to be contacted.

Clinical assessment of HBsAg positive patients conducted by the clinician involved in LTC included liver function testing, HBV DNA quantification, Hepatitis B e antigen (HBeAg) and Hepatitis D (HDV) serology testing. Routine clinical information about the patients disease awareness, prior LTC, treatment, monitoring and HCC screening were collected. Non‐invasive fibrosis assessment with aspartate aminotransferase to platelet ratio index (APRI)^14^ and transient hepatic elastography (FibroScan®)^15^ was performed where possible. Patients were considered to have cirrhosis if their hepatic elastography score was greater than 12.5 kPa without other explanation, or if clinical and/or radiological evidence of cirrhosis was present, as assessed by two independent hepatologists. This was defined as examination features of cirrhosis or portal hypertension or radiologic features of a nodular liver contour and/or portal hypertension. Previous HCC screening activity was assessed for each patient and screening arranged if indicated and had not been previously performed. HCC screening was recommended according to standard for HBsAg positive patients with cirrhosis, HDV coinfection, a first‐degree relative with a history of HCC or those otherwise at increased risk (Asian or African men over 40 years, Asian women over 50 years).^12^ AVT was recommended if indicated and commenced where possible.^12^ Patients were followed up to 18 months following screening.

### 2.2 HBsAg testing rates before and during SEARCH pilot (OB)

We analysed HBV testing rates in Overseas Born patients who presented to the emergency department during the SEARCH pilot and compared this with a period when SEARCH was not operational.

### 2.3 SEARCH pilot cost analysis

All programme costs from the time of ED presentation until the detection of HBsAg positive patients were calculated. Costs were divided into two groups—(i) the direct or actual cost of HBsAg testing and (ii) the indirect costs (programme administrative costs and other laboratory costs). All costs were in Australian dollars ($AUD) in 2018. In several cases, the exact cost of inputs was known (translation and printing costs for patient information, pathology request forms, quality control costs etc.), priced on the actual amount paid. The actual price of HBsAg testing was determined by the total annual cost of the reagents divided by the number of tests performed. Capital costs were not included. Analysers within the hospital are used for routine clinical purposes. For staff time, a human resource study was conducted by real‐time monitoring of staff workflow during the pilot. The average time taken to perform the specific tasks was assessed (with upper and lower time duration) and salary cost was calculated.

### 2.4 Ethics for retrospective analysis of SEARCH pilot

Ethics approval for this retrospective analysis of testing of the OB patients was obtained from the South Western Sydney Local Health District Human Ethics Research Committee (approval: 2019/ETH00656).

### 2.5 Sample size and statistical analysis for analysis of SEARCH pilot

A sample size calculator determined that a minimum of 4706 patients were required to detect a prevalence of 2% with a precision of .4%, 95% confidence intervals. Analyses were conducted using the IBM® Statistical Package for the Social Sciences version 25.0.

## 3 RESULTS

### 3.1 Efficacy of automated testing in the ED

**FIGURE 1:**
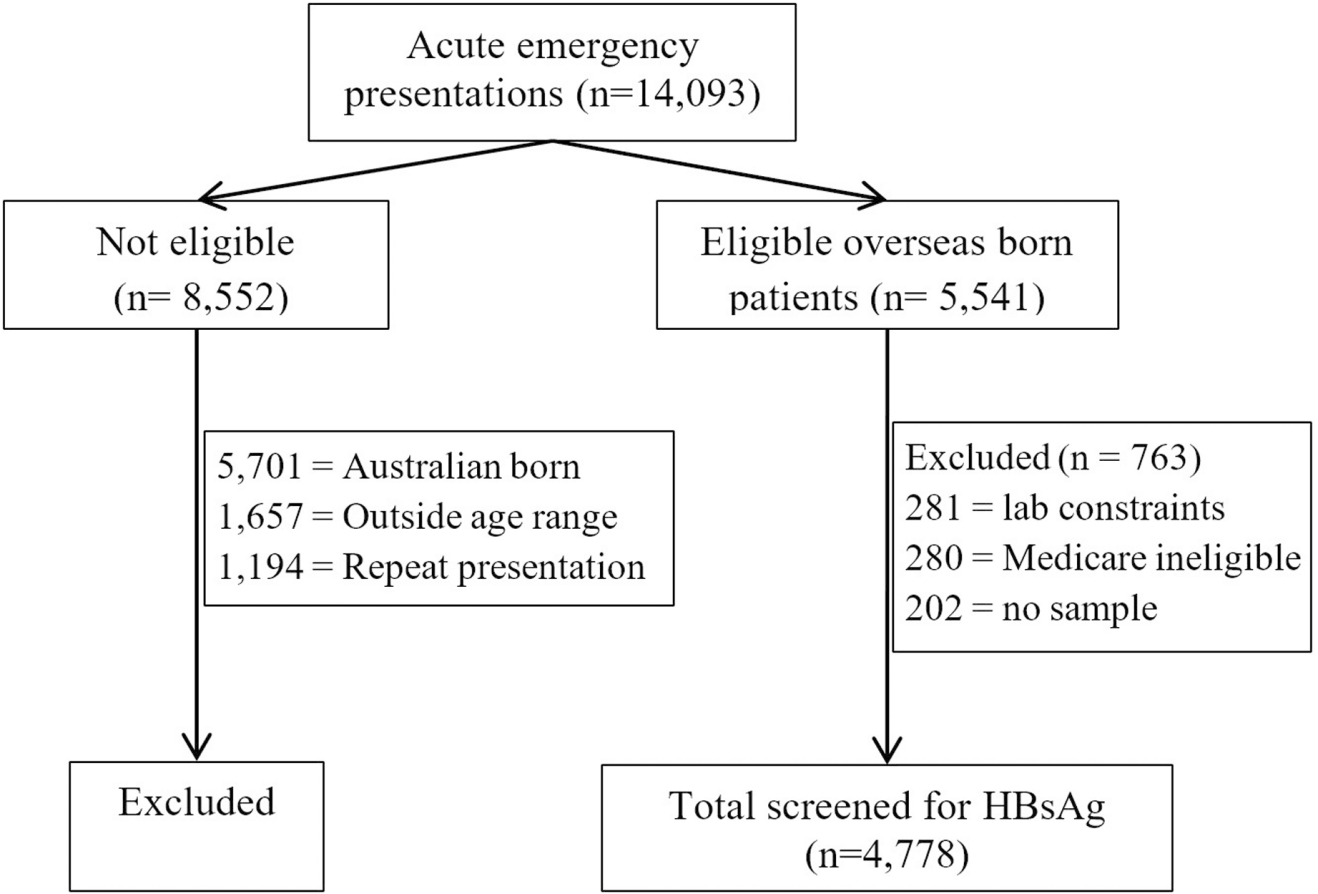
Enrolment flowchart. HBsAg, hepatitis B surface antigen.

Between July 2018 to February 2019, the SEARCH pilot programme identified 5541 eligible OB patients from 14 093 consecutive ED presentations utilising electronic screening of admission demographics. Of these, 4778 (86%) were successfully tested for HBV using the add‐on system introduced for the pilot. Fourteen per cent were not screened largely due to laboratory constraints (Figure 1). Characteristics of screened patients have been presented in a previous report of HCV Ab testing conducted at the same time.^16^ No patients withdrew consent for hepatitis testing during this period. Those with positive results who were linked to care reported satisfaction with and gratitude for the SEARCH service.

The testing rate in the OB population prior to the SEARCH pilot and reliant on clinician initiative alone was examined. In 471 consecutive (age standardised) OB patients presenting to ED in January 2020, only nine (1.9%) patients were tested for HBV, much less than during SEARCH (*p* < .0001). Notably, at least nine of the OB patients had an additional risk factor for testing such as being PWID. Despite this none of them were tested for HBV.

### 3.2 Characteristics of HBsAg positive patients identified through SEARCH

There were 108 patients (2.3%) who tested HBsAg positive of the 4778 patients screened. The highest rate occurred in patients from Tonga (14%), Vietnam (9%), Laos (6%) and China (5%) (Table 1). Patients were from 30 different countries with 63% speaking a primary language other than English. There was a male predominance (57%), with a mean age of 56.3 years (SD 12.2). Only 7% of positive patients were admitted under gastroenterology and 45% (49/108) were discharged directly from ED (Table 2).

**TABLE 1:** Hepatitis B prevalence by country of birth^a^

	High (>5%)	Medium (1%–5%)		Low (<1%)
HBsAg +ve	Tonga (14)	Cambodia	Turkey	Iraq
	Vietnam (9)	Philippines	Poland	Lebanon
	Laos (6)	Macedonia	Bangladesh	Fiji
	China (6)	New Zealand	Portugal	
	Samoa (5)	Greece	Malaysia	
		Syria		
		Egypt		

Abbreviations: HBsAg, hepatitis B surface antigen.

^a^ Countries with <25 screened patients or no positive patients were excluded.

**TABLE 2:** Reason for ED presentation and speciality of admission

Reason for ED presentation (%)		Admitting team (%)	
Abdominal pain	15 (14)	Emergency medicine^a^	49 (45)
Sepsis	14 (13)	General medicine	10 (9)
Chest pain	13 (12)	Surgery	7 (7)
Neurological symptoms	10 (9)	Cardiology	7 (7)
Shortness of breath	8 (7)	Gastroenterology	7 (7)
Mental illness	7 (7)	Neurology	6 (6)
Musculoskeletal injury	7 (7)	Psychiatry	5 (5)
Syncope	6 (6)	Respiratory medicine	5 (5)
Abnormal laboratory results	6 (6)	Haematology	3 (3)
Trauma	5 (5)	Gynaecology	1 (1)
Dizziness	3 (3)	Other internal medicine	8 (7)
Gastrointestinal bleeding	2 (2)		
Abnormal liver function	1 (1)		
Other	11 (10)		

^a^ Patients discharged from the emergency department.

Twenty patients (19%) were classified as new HBV diagnoses of whom the majority were male (70%). Antiviral treatment was indicated in 8/20 including five patients with a new diagnosis of cirrhosis.

Eighty‐eight patients had a previous or known diagnosis of HBV. Their HBV was actively managed by a specialist in 42 (48%) and by a primary care physician in eight (9%). Nine (10%) had never sought medical care for HBV despite being aware of their diagnosis and 29 (33%) had been LTFU prior to re‐diagnosis as a result of the SEARCH pilot.

Hepatitis D (HDV) testing was available in 52/108 (48%) of patients. Out of those available, only 2 (4%) had HDV coinfection.

Cirrhosis was identified in 21/108 (19%) patients—Child Pugh (CP) A, B and C in 14, 6 and 1 respectively (Table S1). Their care was managed by a specialist in 10. Five were not under care despite a known HBV diagnosis, one had never sought medical care for HBV and five had new diagnoses of both HBV and cirrhosis.

Patients with cirrhosis were older (62 vs. 55 years, *p* = .017) and more likely to be male (86% vs. 51% *p* = .003). The admitting team was more likely to be gastroenterology in patient with cirrhosis, although that was still the minority (24% vs. 2%, *p* = .009) (Table 3). Cirrhotic patients were less likely to be on AVT compared to non‐cirrhotic patients in whom treatment was indicated (43% vs. 74%, *p* = .019). There was no difference in the rates of diagnosis, medical follow‐up, DNA monitoring or HCC screening between cirrhotic and non‐cirrhotic patients (Table 3).

**TABLE 3:** Characteristics ofHBsAg positive overseas born patients and analysed according to presence/absence of cirrhosis

	All (*n* = 108) (%)	Cirrhosis (*n* = 21) (%)	No cirrhosis (*n* = 87) (%)	*p* value
Mean age (years) (±SD)	56.3 (12.2)	62.0 (10.0)	55.0 (12.3)	**.017**
Male gender (%)	62 (57)	18 (86)	44 (51)	**.003**
Median BMI^a^ (IQR)	26 (22–30)	26 (23.0–35.5)	26 (21.5–29.0)	.246
Active PCP	89 (82)	19 (91)	70 (81)	.356
Interpreter required	41 (38)	10 (48)	31 (36)	.310
Discharged from ED	49 (45)	6 (29)	43 (49)	.085
Admitted under gastroenterology	7 (6)	5 (24)	2 (2)	**.009**
Admitted under another service	52 (485)	10 (48)	42 (48)	
Previously diagnosed	88 (81)	16 (76)	72 (83)	.534
Seeing a doctor for hepatitis B	50 (46)	10 (48)	40 (46)	.892
HBV DNA level performed in the previous 12 months^b^	49 (57)	11 (69)	38 (53)	.551
History of HCC	7 (6)	7 (33)	0 (0)	**<.001**
HCC screening or surveillance in last 8 months^c^	19 (24)	8 (38)	11 (19)	.086
Receiving antiviral treatment when indicated	35 (63)	9/21 (43)	26/35 (74)	**.019**
Appropriate overall care	30 (28)	8 (38)	22 (25)	.195
Median transient elastography (kPa) (IQR)	5.5 (4.2–8.2)	24.0 (14.0–30.5)	4.9 (4.0–6.2)	**<.001**
Median ALT (U/L) (IQR)	27 (20–42)	46.0 (24.0–93.5)	25.5 (18.0–37.0)	**<.001**
Median AST (U/L) (IQR)	29 (24–43)	53.0 (37.5–128.5)	27.0 (22.8–34.5)	**<.001**
Median PLT count (×10^9^/L) (IQR)	207 (167–266)	158 (103.5–203.5)	221 (177.3–227)	**<.001**
Hepatitis B e antigen positive^d^	10 (10)	7 (33)	3 (4)	.376
Hepatitis D antibody positive^e^	2 (4)	2 (13)	0 (0)	**.001**
Hepatitis C antibody positive	5 (5)	3 (14)	2(2)	**.049**

Note: *P* value in bold when signficant <0.05 (Chi square)

Abbreviations: ALT, alanine aminotransferase; AST, aspartate aminotransferase; BMI, body mass index; HCC, hepatocellular carcinoma; IQR, interquartile range; PCP, primary care provider; PLT, platelet.

^a^ Not available in 15/108 cases.

^b^ Newly diagnosed excluded.

^c^ Only patients in whom HCC is indicated included in this analysis (57 non‐cirrhotics).

^d^ Not available in 6/108.

^e^ Not available in 56/108 cases.

### 3.3 HBV care milestones prior to LTC through SEARCH

HBV care milestones were assessed in the 88 patients with a known prior HBV diagnosis. Milestones evaluated were annual HBV DNA quantification, HBV treatment and HCC surveillance where indicated.

HBV DNA quantification had been performed in the preceding 12 months (Table 4) in 49 of the 88 with a known diagnosis of HBV (56%). Thirty‐five patients (40%) were on AVT (Entecavir = 24, Tenofovir = 8 and Lamivudine = 3). Fifty‐three patients were not taking AVT including, seven patients with cirrhosis and six patients with *HBeAg negative hepatitis* (ALT elevated, i.e. immune escape).

**TABLE 4:** HBV care milestones prior to LTC through SEARCH

Metric	(*n* = 88)
Seeing a doctor about HBV	50/88
Receiving HBV DNA quantification within last 12 months	49/88
Receiving HCC screening (where indicated)^a^	13/56
Receiving antiviral therapy (where indicated)	35/48
Receiving complete guideline‐directed care	30/88

Abbreviations: HCC, hepatocellular carcinoma.

^a^ Patients with a history of HCC excluded.

Participation in an HCC screening programme was indicated in 62/88 patients based on current guidelines as previously described.^12^ Appropriate screening occurred in only 13 (23%) of 56 patients (6 patients with prior or known HCC were excluded from this analysis as they were already being managed within a liver unit).

**FIGURE 2:**
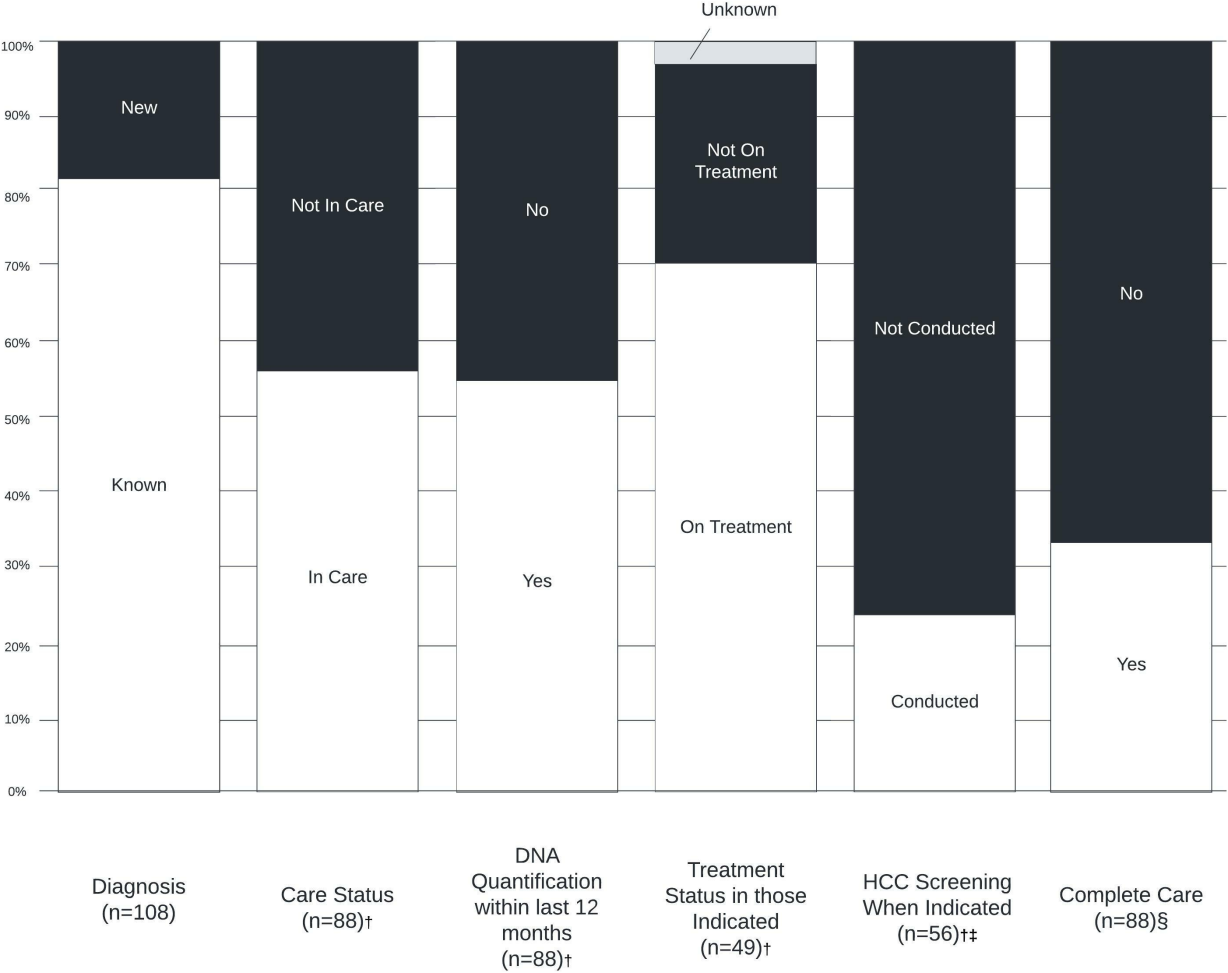
Hepatitis B care milestones. HCC, hepatocellular carcinoma. ^†^Patients newly diagnosed (*n* = 20) were excluded from these analyses. ^‡^Patients with known or prior HCC (*n* = 6) were excluded from this analysis. ^§^Complete care definition: appropriate HBV DNA quantification, treatment and HCC screening.

Combining all this data regarding the 88 with a known diagnosis of HBV identified by SEARCH, only 30 (34%) were receiving complete guideline‐based HBV care (Figure 2). This metric was not different between cirrhotic and non‐cirrhotic patients (38% vs. 25%, *p* = .195).

### 3.4 Linkage to care and treatment following SEARCH

Forty‐eight of 58 (83%) patients were successfully linked to specialist or primary care after SEARCH testing. Twenty were new diagnoses and 38 known diagnoses but not currently receiving HBV care. Five patients were unable to be contacted, three patients failed to attend their appointments and two had died.

Based on assessment through SEARCH, treatment was indicated in a further 21 patients, not on treatment (8 new diagnosis and 13 known diagnoses). This included 12 patients found to have current or previously documented cirrhosis and 9 non‐cirrhotic patients; 8 with *HBeAg negative hepatitis* (immune escape) and 1 with *HBeAg negative infection* (immune control) who was on immunosuppressive medication. After LTC, 19 of the 21 (90%) with an indication for AVT were commenced on treatment. One patient was newly diagnosed with HCC.

### 3.5 SEARCH cost analysis

The overall cost of the HBsAg screening pilot for 4778 patients was calculated to be $34 643.35 (Table 5) comprising $6928.10 direct costs for actual HBsAg assay and $27715.25 indirect costs (administrative, staff and laboratory). The overall cost per patient tested was $7.21 and the cost per HBsAg positive patient identified was $320.77. If SEARCH utilised a system of prospective automation of HBsAg test ordering, removing the step of manual ordering and specimen retrieval, $25 071.24 of the indirect costs could be saved. The total cost would then be only $9572.11 to screen 4778 patients or $2.00 per patient tested and $88.63 per HBsAg positive patient in this cohort.

**TABLE 5:** Detailed costs of HBsAg testing of 4778 emergency department patients

Item	Cost	Lower estimate	Upper estimate	Source and explanation
Patient education materials	$1781.55	—	—	Supplier receipt
Request form cost^a^	$477.80	—	—	Supplier receipt ($.1 per request × 4778)
Manual ordering of tests^a^	$5290.32	$3526.88	$7053.76	90 min per day (range 60–120 min) 134 weekdays programme ran; hourly rate of pay—$26.32
Laboratory staff manually retrieving samples^a^	$19303.12	$10 100	$30 300	6 min per sample (range 3–9 min) × 4778 samples Hourly rate of pay—$41.40
HBsAg testing	$6928.10	—	—	Actual laboratory cost ($1.45 × 4778)
Confirmation testing for positive and indeterminate results	$814.68	—	—	Actual laboratory cost ($6.57 × 124 patients)
HBsAg assay quality control	$47.78	—	—	Actual laboratory cost ($.01 per test × 4778)
Total cost	$34 643.35	$23 228.35	$46 058.35	

^a^ Areas of potential cost saving with further automation of testing.

## 4 DISCUSSION

This study demonstrates the efficacy and utility of automated routine ED screening for HBV, with nearly 90% of those eligible successfully tested, compared with a testing rate of 1.9% in a non‐SEARCH period. This method operates even when competing healthcare priorities and barriers exist.

Chronic HBV infection was found in 2.3% of the screened OB population with very high prevalence in some subgroups. A significant proportion were either unaware of their infection (19%) or not receiving follow‐up (35%). Care for those with known HBV was incomplete, with only 28% receiving the recommended standard of care. This study highlights a large unmet need in HBV care.

There was a low rate of HDV testing performed (48%) in this study compared to guideline recommendations of routine testing in all patients.^13^ Of those tested, only 2/52 (4%) were found to have HBV/HDV coinfection in line with the low prevalence of HDV reported in Australia and the Asia‐Pacific region.^17^, ^18^

In line with the 2030 WHO viral hepatitis elimination goals, Australia aims to increase HBV diagnoses, improve rates of care and increase AVT by 2022.^5^ Modelling demonstrates these national targets will not be met unless there are considerable increases in testing and treatment.^19^ HBV screening in high‐risk populations has been demonstrated to be cost‐effective^8^, ^9^, ^10^ however implementation remains a barrier, with some calling for universal rather than risk‐based screening.^11^ Despite existing recommendations for screening of OB patients, this population remains difficult to engage, due to barriers including language, as demonstrated by the fact that 38% of patients in the SEARCH service required an interpreter.

Screening in the ED is advantageous for many reasons as it: (i) reaches individuals not otherwise engaged in healthcare; (ii) reaches difficult to access patients such as those from CALD backgrounds; (iii) tests people who may not otherwise attend their PCP; (iv) may be easier to implement than screening in primary care where their broad range of practitioners and models of care prohibit standardisation and (v) allows direct linkage to specialist care.

Previous studies of ED screening for blood‐borne viruses (BBV) have been limited by low uptake rates given staff were required to collect additional samples for BBV testing.^20^, ^21^ In contrast, the SEARCH service tested for HBV using previously collected biochemistry samples. In our analysis, patients with HBV presented for reasons unrelated to viral infection, most commonly chest pain, sepsis and abdominal pain. Testing for viral hepatitis risk without a systematic programme was poor, as evidenced by our control group analysis.

Studies of patients wishes report that if hospitals automatically tested for BBVs, 75% would prefer to be tested without knowing, then to not be tested at all.^22^ Staff feedback about such automated processes suggests facilitated screening and elimination of the need for manual assessment of risk factors is supported.^23^ Burdening ED staff with additional work is recognised as an obstacle to BBV testing.^24^

Following ED presentation, 87% of patients were successfully linked to care, 29% of patients required AVT of whom over half were cirrhotic. These findings compliment results for HCV testing in the ED, previously reported.^16^ High LTC is critical for a successful testing programme. This cohort was successfully engaged and contrasts other reports where LTC was poor.^25^ Differences in cohorts rather than infrastructure probably explain this difference. An ‘all comer’ approach offered by SEARCH may more realistically reflect the LTC outcomes.

Benefits of testing included HBV re‐diagnosis in addition to new diagnoses, as well as facilitating LTC. Guideline‐based HCC screening, treatment and monitoring had not been performed in many patients.^26^, ^27^

We found that the cost of HBsAg testing within the SEARCH pilot was relatively inexpensive—$7.25 per patients tested and $320.77 per HBsAg positive patient detected. The costs of testing would be reduced to $2.00 per patient tested if indirect costs were reduced by improvements in the testing algorithm.

### 4.1 Limitations

There are some limitations to this analysis. There was high prevalence of HBV in our overseas born ED population (2.3%) compared to estimated prevalence of 0.9% in the general Australian population. If universally adopted, the cost per HBsAg positive patient found would be higher due to the lower expected prevelance. The utility of SEARCH will be influenced by the local patient populations and the rates of new diagnosis and patients lost to follow‐up by the local level of specialist and PCP support. ED cohorts enriched with older patients with higher rates of HBV, when vaccination rates in their birth country may be more relevant than those with numbers of younger OB patients.^28^ The strategy would benefit from evaluation in other sites with a different prevalence of HBV priority populations and with different tertiary clinic supports. Particularly we acknowledge that our ED is surrounded by a community with lower socio‐economic status, poorer health literacy and more likely to have been born in regions with low infant vaccination for HBV at the time of their birth.

The cost analysis only assessed the actual costs of HBsAg testing for ED patients in a public hospital, not the charges that might be applied in a private setting. We also did not analyse the costs of follow‐up and management of HBsAg positive patients which was incorporated into standard clinical care within our institution. Costs may vary between laboratories and may be more expensive to perform in smaller laboratories. The cost per HBsAg positive patient found is dependent upon the prevalence of HBV in the community who are tested though remains a cheap test, so this is unlikely to make the programme unaffordable.

This SEARCH pilot required manual add‐on ordering of HBV testing followed by retrieval of the biochemistry specimen. Full automation of this process is under development so that testing can be linked to initial order, avoiding the specimen retrieval step. This will reduce labour required and be more cost‐effective.

## 5 CONCLUSION

This novel pilot screening service of routine automated and universal HBV testing in the Overseas Born population, was effective in HBV diagnoses, re‐diagnoses and achieving linkage to care.

## FUNDING INFORMATION

There was no funding source for this publication.

## CONFLICT OF INTEREST

The authors declare that there is no conflict of interest.

## ETHICS STATEMENT

Ethics approval for this research was obtained.

## Supporting information


**Appendix S1** Supporting Information.

## Data Availability

The data used to support the findings of this study are available from the corresponding author upon request.
