# Supplementary material for: Routine screening of emergency admissions at risk of chronic hepatitis (SEARCH) identifies and links hepatitis B cases to care
Source: Liver Int. 2022 Sep 14;43(1):60–8. doi: 10.1111/liv.15414 (PMC10087472; doi:10.1111/liv.15414)
Supplement: Supplementary file 1 — Appendix S1 Supporting Information. [file LIV-43-60-s001.docx]

Supplementary Table – HBsAg positive patients with cirrhosis

| Case number | | Age | Child Pugh Score | Method of cirrhosis diagnosis | Transient elastography  (TE) (kPa) | Platelet count  (x 10^9^/L) | Portal hypertension† | HBV diagnosis | Cirrhosis diagnosis | Presence of HCC | Previously on treatment | APRI score |
| --- | --- | --- | --- | --- | --- | --- | --- | --- | --- | --- | --- | --- |
| HBV 1 | | 69M | B9 | Clinical | 8.8‡ | 80 | No | New | New | No | No | 0.5 |
| HBV2 | | 69M | A6 | Clinical | 14.5 | 173 | No | New | New | No | No | 5.0 |
| HBV15 | | 65M | B9 | Clinical | 50 | 81 | Varices | Known | Known | No | Yes | 1.6 |
| HBV24 | | 42M | B9 | Clinical | 39 | 60 | Varices | Known | Known | Yes | Yes | 1.6 |
| HBV37 | | 49M | A5 | Clinical | 13.5 | 42 | No | Known | Known | No | No | 2.3 |
| HBV52 | | 65M | A5 | Radiology | Nil | 165 | No | Known | Known | Yes | Yes | 0.6 |
| HBV53 | | 71F | A6 | Clinical | 30.7 | 71 | Varices | Known | Known | No | No | 2.8 |
| HBV64 | | 64M | A5 | Clinical | Nil | 200 | No | Known | Known | Yes | Yes | 0.3 |
| HBV65 | | 69F | A6 | Clinical | Nil | 158 | Varies | Known | Known | Yes | Yes | 1.3 |
| HBV66 | | 38M | A5 | Radiology | Nil | 285 | Varices | Known | New | No | No | 0.2 |
| HBV70 | | 60M | C12 | Clinical | 75 | 247 | Splenomegaly | New | New | New diagnosis | No | 0.5 |
| HBV71 | 62M | | A6 | Clinical | 16 | 264 | Varices | Known | Known | Yes | Yes | 0.3 |
| HBV80 | 74M | | A5 | Clinical | Nil | 143 | No | Known | Known | No | Yes | 0.3 |
| HBV87 | 58M | | A5 | Clinical | 27.9 | 130 | Varices | New | New | No | No | 1.5 |
| HBV89 | 63M | | A5 | Clinical | 21 | 197 | No | Known | New | No | No | 0.3 |
| HBV90 | 73F | | A5 | Radiology | Nil | 207 | No | New | New | No | No | 0.5 |
| HBV93 | 59M | | A5 | Clinical | Nil | 186 | No | Known | Known | No | Yes | 1.4 |
| HBV97 | 54M | | A6 | Biopsy | 22 | 252 | No | Known | Known | No | No | 0.4 |
| HBV107 | 52M | | B9 | Clinical | Nil | 144 | Varices | Known | Known | Yes | Yes | 3.1 |
| HBV110 | 72M | | A5 | Clinical | 30 | 126 | Ascites | Known | Known | Yes | Yes | 2.4 |
| HBV111 | 72M | | B9 | Clinical | 26 | 142 | Ascites | Known | Known | No | No | 16.5 |

† Portal Hypertension defined as the presence of ascites or gastrointestinal varices at upper endoscopy.
‡ FibroScan performed 2 years prior to SEARCH encounter. HBV + HDV coinfection leading to rapid disease progression.
